# A Case of Bordetella bronchiseptica Bacteremia in a Patient With Decompensated Liver Cirrhosis

**DOI:** 10.7759/cureus.13938

**Published:** 2021-03-17

**Authors:** Kok Hoe Chan, Susanne O Ajao, Iyad Farouji, Jihad Slim

**Affiliations:** 1 Internal Medicine, Saint Michael’s Medical Center, Newark, USA; 2 Infectious Diseases, Saint Michael’s Medical Center, Newark, USA

**Keywords:** bordetella bronchiseptica, bacteremia, gram negative coccobacillus, decompensated liver cirrhosis

## Abstract

*Bordetella bronchiseptica* is a rare cause of respiratory tract infection in humans, most commonly found in immunocompromised individuals exposed to infected animals. It colonizes the respiratory tract and can lead to infection in dogs, cats, rabbits, and others. In immunocompromised patients, it has been reported to result in life-threatening infections but rarely affects immunocompetent individuals. Here, we are the first to report a case *B. bronchiseptica *bacteremia in a patient with decompensated liver cirrhosis without known animal exposure.

## Introduction

*Bordetella bronchiseptica*, a gram-negative coccobacillus, is associated with infections in animals. Although it is a rare cause of human disease, cases including bacteremia and pneumonia have been reported in immunocompromised hosts [1-4]. Here, we present a rare case of a 63-year-old gentleman who was immunocompromised with liver cirrhosis and developed *B. bronchiseptica* bacteremia. To the best of our knowledge, no cases of *B. bronchiseptica* bacteremia in patients with liver cirrhosis have been reported in the literature.

## Case presentation

A 63-year-old African American male with past medical history of congestive heart failure, pulmonary sarcoidosis, non-alcoholic liver cirrhosis, chronic kidney disease, and hypertension presented with complains of shortness of breath and bilateral lower extremity swelling. He denied abdominal pain, upper respiratory tract symptoms, fever, chills, and rigor. He also denied recent exposure to pets and animals including dogs. Initial vital signs were stable. He was afebrile, normotensive, and saturated at 100% on room air. Physical examination was significant for a markedly distended abdomen, which was soft and non-tender to light and deep palpation. Jugular venous distension was evident with extensive bilateral lower extremity edema with scrotal edema. There was no jaundice on examination. Chest X-ray was unremarkable with no infiltrates and consolidations. Initial laboratory investigation revealed a white count of 4.2 cells/mm^3^ (4.4-11.0 cells/mm^3^), hemoglobin of 10.7 g/dL (13.5-17.5 g/dL), platelet of 188 cells/mm^3^ (150-450 cells/mm^3^), lactic acid of 2.3 mmol/L (0.0-2.0 mmol/L), creatinine of 1.7 mg/dL (0.6-1.2 mg/dL), B-type natriuretic peptide of 823.71 pg/mL (0.0-100.0 pg/mL), protein of 8.2 g/dL (6.4-8.4 g/dL), and albumin of 2.6 g/L (3.5-4.5 g/L). Liver function tests were mildly elevated with an aspartate aminotransferase of 43 U/L (10-36 U/L), alanine aminotransferase of 20 U/L (9-46 U/L), and alkaline phosphatase of 134 U/L (40-115 U/L). Total bilirubin was within normal limit. Coagulation profile showed slightly prolonged international normalized ratio of 1.46.

He was admitted and managed for decompensated liver cirrhosis with diuresis to relieve the edema. He had undergone similar admissions in the past and also had multiple paracentesis done, the most recent was a month earlier, for symptomatic relief. A large-volume paracentesis was done during this hospitalization with the removal of 10 L of peritoneal fluid. Following paracentesis, he became hypotensive despite resuscitative efforts with appropriate albumin. Peritoneal fluid analysis revealed a white blood cell count of 72/mm^3^, 49% lymphocyte differential, 44% neutrophil differential, protein of 2.6 g/dL, lactate dehydrogenase of 91 U/L, and glucose of 113 mg/dL. This analysis was consistent with a diagnosis of portal hypertension likely as a result of his liver cirrhosis, and spontaneous bacterial peritonitis was unlikely. No organism was isolated from peritoneal fluid cultures and gram stain showed no organism.

**Figure 1:**
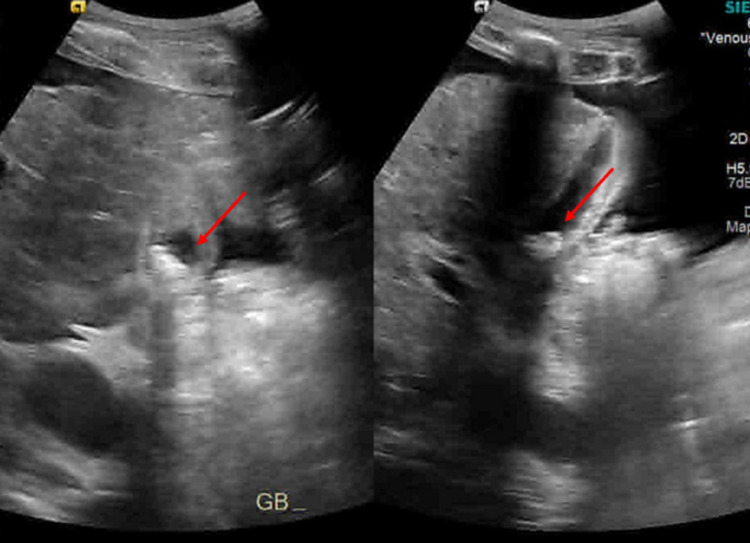
Right upper abdomen showed contracted gallbladder with gallstones, with no evidence of cholecystitis: transverse (left) and longitudinal (right) views.

In the setting of his persistent hypotension, sepsis was considered, blood cultures were drawn, and meropenem was empirically started. Ultrasound of the abdomen was done to look for a source and showed moderate ascites with a contracted gallbladder with no evidence of cholecystitis (Figure 1).

**Figure 2:**
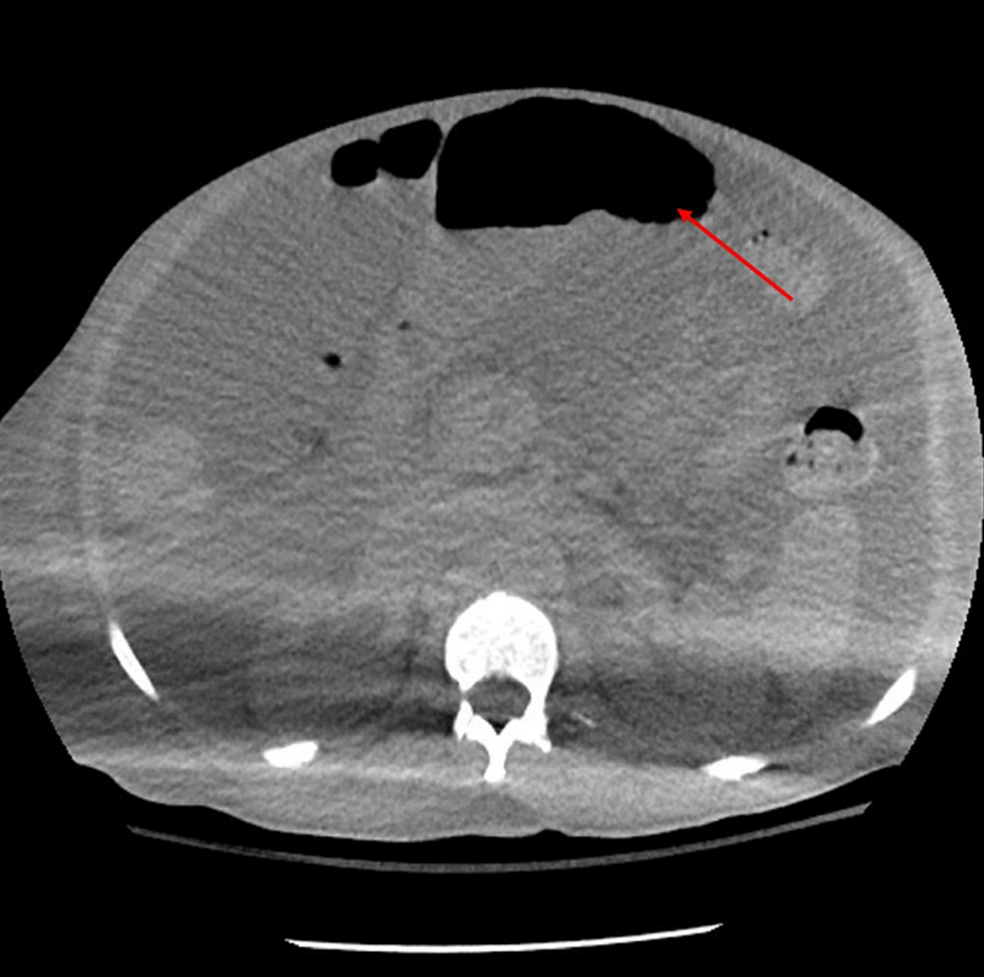
CT of the abdomen pelvis showed huge amount of ascites without evidence of intra-abdominal abscess. CT, computed tomography

Computed tomography of the abdomen was also done which showed cirrhosis and cholelithiasis with no evidence of intra-abdominal abscess (Figure 2).

A hepatobiliary iminodiacetic acid scan was done which revealed no biliary obstruction but delayed visualization of the gallbladder. Surgery was consulted and in the absence of clinical signs of cholecystitis with his multiple comorbidities, elective cholecystectomy was postponed, and ongoing antibiotic management was advised. *B. bronchiseptica* was isolated from blood cultures (Table 1).

**Table 1:** Culture and sensitivity of Bordetella bronchiseptica.

Bordetella bronchiseptica	
Amikacin	<= 16 Susceptible
Cefepime	<= 8 Susceptible
Cefotaxime	8 Susceptible
Ceftazidime	4 Susceptible
Ceftriaxone	<= 8 Susceptible
Ciprofloxacin	<= 1 Susceptible
Gentamicin	<= 4 Susceptible
Meropenem	<= 4 Susceptible
Piperacillin + tazobactam	<= 16 Susceptible
Tetracycline	<= 4 Susceptible
Tobramycin	<= 4 Susceptible
Trimethoprim + sulfamethoxazole	<= 2/38 Susceptible

**Figure 3:**
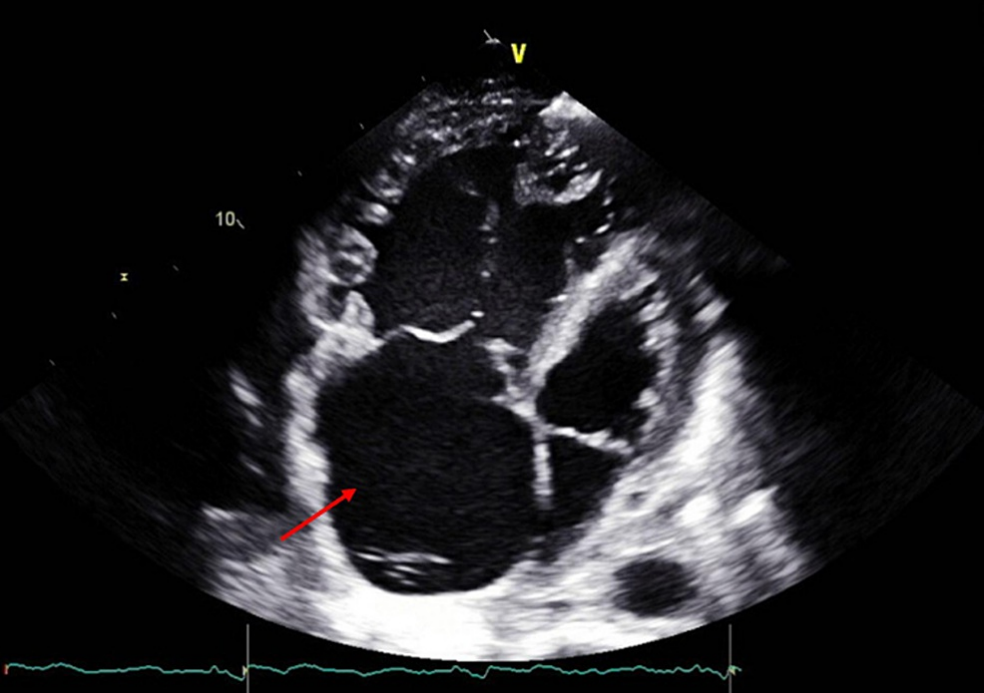
Echocardiogram four-chamber views showing severely dilated right atrium and right ventricle, with no evidence of vegetations.

Echocardiogram was performed and did not show any vegetations (Figure 3).

Meropenem was stopped and based on a review of the available literature, we decided to start the patient on doxycycline for the bacteremia. Blood pressure stabilized after 48 hours and repeat blood cultures were negative. The patient tolerated doxycycline and was discharged with a seven-day course of oral doxycycline.

## Discussion

*Bordetella* are gram-negative bacterial pathogens of mammals and birds. The most common species are *B. pertusis, B. parapertusis*, and *B. bronchispetica* [5]. *B. pertussis*, the causative agent of whooping cough, is a human-adapted variant of *B. bronchiseptica. B. bronchispetica* can colonize the upper respiratory tract of different animals [6]. It is very rare to isolate *B. bronchispetica *from human beings despite their frequent exposure to animals commonly colonized with this organism, including mice, dogs, cats, poultry, and livestock such as pigs. Most of the reported cases of *B. bronchispetica *were in severely immunocompromised patients [7]. It is believed that *Bordetella *species enter the respiratory tract through aerosol droplets [8]. In comparison to other pathogens, *Bordetella *have the ability to adhere efficiently to healthy, rapidly beating ciliated epithelial cells, where they begin to proliferate and produce factors that counteract host defenses, which prevents their elimination [9].

*B. bronchiseptica* is pathogenic in mammalian species and produces an endotoxin that is similar in chemical composition and physiological effects to those of other gram-negative microorganisms [10]. The bacterium can adhere to respiratory epithelial cells by using fimbriae and filamentous hemagglutinins. It invades respiratory epithelial cells and alveolar macrophages and diminishes the overall bactericidal ability of these cells by producing the enzyme adenylate cyclase. These virulence factors enable the organism to successfully colonize the respiratory tract [11].

All *B. bronchiseptica* infections that have been reported in the literature were in immunocompromised patients who got the zoonotic infection through animal contact. *B. bronchiseptica* infections have been reported in patients with human immunodeficiency virus, hematological malignancy, or those on immunosuppressive medications [3,4]. Patients may present with a wide range of respiratory symptoms, ranging from asymptomatic to severe [3,4]. Patients infected with *B. bronchiseptica* typically present with classic symptoms of pneumonia, and in some cases, present with acute sinusitis and bronchitis. Our patient had extensive imaging done that did not show any abscesses in the body that may harbor this organism.

Patients with liver cirrhosis often have acquired immunodeficiency and systemic inflammation with increased risk or susceptibility to bacterial infections. This condition is recognized as cirrhosis-associated immune dysfunction [12]. Patients with liver cirrhosis not only have reduced number of neutrophils due to its sequestration by the enlarged spleen but also have been reported to have neutrophil dysfunction with defective phagocytosis of opsonized bacteria [13,14]. Nonetheless, to the best of our knowledge, there are no prior case report or studies on *B.* *bronchiseptica* in liver cirrhosis. We propose that the defective neutrophils may be the cause of bacteremia in our immunocompromised patient.

The diagnosis of *B. bronchiseptica* depends on positive cultures or polymerase chain reactions in patients with exposure to animals [15]. Blood cultures and bronchoalveolar lavage colony more than 104, in case of pneumonia, can help to determine whether *B. bronchiseptica* is a real infection or just airway colonization [16]. Regarding treatment, *B. bronchiseptica* can be treated as gram-negative non-fermentative pathogen [17]. Nonetheless, Schipper et al., despite the fact that *B. bronchiseptica* is an extracellular pathogen, the organism can invade and persist in eukaryotic cells, such as phagocytes and even epithelial cells; hence, it is very important to choose a medication with good intracellular penetration to prevent recurrent and chronic infections [18]. Various antibiotics can be used including aminoglycosides, quinolones, anti-pseudomonal penicillins, tetracycline, trimethoprim-sulfamethoxazole, imipenem, or a combination of erythromycin, ciprofloxacin and rifampin depending on the susceptibility [19,20]. Due to the rarity of this infection, there are no clear guidelines regarding the duration of the treatment. To our knowledge, we are presenting the first case in literature. Our patient had liver cirrhosis and presented with *B. bronchiseptica* bacteremia without any history of animal exposure and respiratory symptoms.

## Conclusions

*B. bronchiseptica* is a gram-negative coccobacillus found commonly in the upper respiratory tract of animals. It can be transmitted to human beings causing zoonotic infection that may lead to life-threatening infection in those with underlying debilitation or impaired immunity (e.g., patients with neutropenia, diabetes, malnutrition, or transplant patients). We presented a very unique and rare case of liver cirrhosis patient who presented with *B. bronchiseptica *bacteremia without any respiratory symptoms and without direct exposure to animals. Due to the rarity of this case, there was no consensus guidelines for the treatment choice and duration. With the limited literature available, we treated the patient with doxycycline for seven days.
